# Effectiveness of Continuous Endotracheal Cuff Pressure Control for the Prevention of Ventilator-Associated Respiratory Infections: An Open-Label Randomized, Controlled Trial

**DOI:** 10.1093/cid/ciab724

**Published:** 2021-08-22

**Authors:** Vu Quoc Dat, Lam Minh Yen, Huynh Thi Loan, Vu Dinh Phu, Nguyen Thien Binh, Ronald B Geskus, Dong Huu Khanh Trinh, Nguyen Thi Hoang Mai, Nguyen Hoan Phu, Nguyen Phu Huong Lan, Tran Phuong Thuy, Nguyen Vu Trung, Nguyen Trung Cap, Dao Tuyet Trinh, Nguyen Thi Hoa, Nguyen Thi Thu Van, Vy Thi Thu Luan, Tran Thi Quynh Nhu, Hoang Bao Long, Nguyen Thi Thanh Ha, Ninh Thi Thanh Van, James Campbell, Ehsan Ahmadnia, Evelyne Kestelyn, Duncan Wyncoll, Guy E Thwaites, Nguyen Van Hao, Le Thanh Chien, Nguyen Van Kinh, Nguyen Van Vinh Chau, H Rogier van Doorn, C Louise Thwaites, Behzad Nadjm

**Affiliations:** 1 Oxford University Clinical Research Unit, Wellcome Trust Africa Asia Programme, Vietnam; 2 Department of Infectious Diseases, Hanoi Medical University, Hanoi, Vietnam; 3 Hospital for Tropical Diseases, Ho Chi Minh City, Vietnam; 4 National Hospital of Tropical Diseases, Hanoi, Vietnam; 5 Trung Vuong Hospital, Ho Chi Minh City, Vietnam; 6 Department of Microbiology, Hanoi Medical University, Hanoi, Vietnam; 7 Centre for Tropical Medicine and Global Health, Nuffield Department of Clinical Medicine, University of Oxford, Oxford, United Kingdom; 8 Department of Adult Critical Care, Guy’s and St Thomas’ NHS Foundation Trust, St Thomas’ Hospital, London, United Kingdom; 9 Medical Research Council The Gambia at The London School of Hygiene & Tropical Medicine, The Gambia

**Keywords:** ventilator, associated pneumonia, hospital acquired pneumonia, continuous cuff pressure control, ventilator, associated respiratory infection

## Abstract

**Background:**

An endotracheal tube cuff pressure between 20 and 30 cmH_2_O is recommended to prevent ventilator-associated respiratory infection (VARI). We aimed to evaluate whether continuous cuff pressure control (CPC) was associated with reduced VARI incidence compared with intermittent CPC.

**Methods:**

We conducted a multicenter open-label randomized controlled trial in intensive care unit (ICU) patients within 24 hours of intubation in Vietnam. Patients were randomly assigned 1:1 to receive either continuous CPC using an automated electronic device or intermittent CPC using a manually hand-held manometer. The primary endpoint was the occurrence of VARI, evaluated by an independent reviewer blinded to the CPC allocation.

**Results:**

We randomized 600 patients; 597 received the intervention or control and were included in the intention to treat analysis. Compared with intermittent CPC, continuous CPC did not reduce the proportion of patients with at least one episode of VARI (74/296 [25%] vs 69/301 [23%]; odds ratio [OR] 1.13; 95% confidence interval [CI] .77–1.67]. There were no significant differences between continuous and intermittent CPC concerning the proportion of microbiologically confirmed VARI (OR 1.40; 95% CI .94–2.10), the proportion of intubated days without antimicrobials (relative proportion [RP] 0.99; 95% CI .87–1.12), rate of ICU discharge (cause-specific hazard ratio [HR] 0.95; 95% CI .78–1.16), cost of ICU stay (difference in transformed mean [DTM] 0.02; 95% CI −.05 to .08], cost of ICU antimicrobials (DTM 0.02; 95% CI −.25 to .28), cost of hospital stay (DTM 0.02; 95% CI −.04 to .08), and ICU mortality risk (OR 0.96; 95% CI .67–1.38).

**Conclusions:**

Maintaining CPC through an automated electronic device did not reduce VARI incidence.

**Clinical Trial Registration:**

NCT02966392.

## Teaser

The results of this randomised controlled trial demonstrate that continuous endotracheal cuff pressure control using an electronic automated device does not reduce the occurrence of ventilator-associated respiratory infection (VARI) in intubated patients compared with intermittent control.

Ventilator-associated respiratory infections (VARIs) are the most common hospital acquired infections (HAI) among intensive care unit (ICU) patients worldwide [1]. VARI encompasses both ventilator-associated pneumonia (VAP) and ventilator-associated tracheobronchitis/tracheitis (VAT). VAT includes tracheobronchitis/tracheitis without evidence of pneumonia [2]. VAP and VAT share manifestations of fever, purulent respiratory secretions, and leucocytosis, but [3], distinguishing these is challenging. Despite guidance against the treatment of VAT with antimicrobials [4], it remains common practice in Vietnam and other settings [5]. In high-income countries (HIC), the incidence density of VARI is 1–10 per 1000 ventilator days [6, 7], and in low- and middle-income countries (LMIC) incidence density is between 3.2 and 56.9 per 1000 ventilator days [8]. VARI are associated with increased length of ICU stay, higher cost of care, and antimicrobial resistant infections [9, 10]. A reduction in VARI incidence would improve patient-centered outcomes and reduce antimicrobial use and thereby potentially slow the development and spread of antimicrobial resistance in the ICU as a whole.

Care bundles deployed to prevent VARI focus on a variety of interventions including strategies to reduce colonization and contamination of the upper airway [11]. Data on the effectiveness of the components of such bundles are lacking, especially in LMIC. Some interventions demonstrated to be beneficial in HIC, such as semirecumbent patient positioning, have failed to demonstrate effectiveness in LMIC [12]. These data are vital to justify resource allocation and improve patient outcomes.

Maintenance of endotracheal cuff pressure at ≥20 cmH_2_O reduces aspiration of contaminated oropharyngeal secretions and may thereby reduce VARI [13]. A conventional approach, advocated in many VARI prevention bundles, involves regular manual checking of cuff pressure with a handheld manometer. Disadvantages include that cuff pressure may only be controlled briefly and that checking pressures takes valuable staff time. This may be particularly pertinent in low-staffed LMIC ICUs, or when staffing is stretched due to high ICU admission rates as seen during the coronavirus disease 2019 (COVID-19) pandemic [14, 15]. Using automated, continuous control of endotracheal cuff pressure could be a preferred approach in such situations as this intervention requires less hands-on time for staff and has little cost in terms of disposable items as compared to innovative endotracheal tubes [16].

Continuous cuff pressure control (CPC) devices were introduced in 1988 [17] and have shown promise in previous studies: a recent meta-analysis of 1 quasi-randomized study (Spain) and 6 open-label RCTs (Spain [1 study], France [2 studies], and China [3 studies]) showed a lower incidence of VAP in the continuous versus intermittent CPC groups (odds ratio [OR] 0.39; 95% confidence interval [CI]: .28–.55) [18–22]. However, there was no difference in the duration of mechanical ventilation or in-hospital mortality. Only 1 study showed a significantly smaller proportion of ICU days on antibiotics in the continuous arm [20]. These are all key indicators of impact, as the diagnosis of VAP is challenging and the assessments were not blinded to allocation [18, 22]. They also, in themselves, form part of the rationale behind efforts to reduce VAP.

Current evidence is insufficient to recommend continuous over intermittent CPC. We therefore conducted a randomised controlled trial with blinded outcome assessment in 3 ICUs in Vietnam to assess the impact of CPC on the incidence of VARI [23].

## METHODS

### Study Design

We conducted an open-label, randomized controlled trial in 3 tertiary referral hospitals: the National Hospital for Tropical Diseases (NHTD) in Hanoi, and the Hospital for Tropical Diseases (HTD) and Trung Vuong Emergency Hospital (TVH) in Ho Chi Minh City. Patients ≥18 years old, who had been mechanically ventilated for <24 hours at the time of screening were eligible for enrolment. Exclusion criteria were previous enrolment in the study, previous intubation within 14 days or known tracheomalacia, tracheal stenosis, or stridor. Patients were stratified by site and diagnosis (tetanus, see the next section).

All patients or their legal guardians provided written informed consent prior to randomization. Ethical approval was obtained through the Oxford Tropical Research Ethics Committee (OxTREC; 26-16), the Hospital for Tropical Diseases (32/HDDD), Trung Vuong Hospital (670/BVTV), and the National Hospital for Tropical Diseases (22/HDDD-NDTU). The study protocol has been published [23].

### Intervention

Eligible patients were randomized to receive manual intermittent CPC (control group) or automated continuous CPC (intervention group). Cuff pressure was recorded in case-record forms 8-hourly in all patients. In the intermittent group, cuff pressure was measured 8-hourly by hand-held manometer (VBM Medizintechnik, Sulz am Neckar, Germany) and if necessary adjusted to the target range at these timepoints. In the intervention group a stand-alone CPC device (701, TRACOE medical, Nieder-Olm, Germany) continuously monitored and adjusted the cuff pressure to maintain the target pressure through inflation/deflation of the cuff. Cuff pressure in both arms was targeted to 25 cmH_2_O, but clinicians could adjust this at their discretion. Participants were followed daily until ICU discharge, at 28 days and 90 days after randomization. Where patients underwent tracheostomy, they continued in the trial, receiving cuff pressure control of the tracheostomy tube cuff through the same method specified by the arm to which they were randomized.

Hospitals followed local infection prevention and control guidelines. For 2 hospitals (HTD and TVH) this included twice daily mouth care with chlorhexidine mouthwash, daily chlorhexidine patient washing, semirecumbent positioning, and suctioning with gloves. NHTD procedures were similar but included thrice daily oral care but no chlorhexidine washing.

No patients were intubated with coated endotracheal tubes, endotracheal tubes designed for subglottic suction, or polyurethane-cuffed tubes. Sites sourced their own endotracheal tubes, which were of a cylindrical (standard) PVC high-volume low-pressure cuff design. None of the study sites employed protocolized spontaneous breathing trials or sedation holds.

Tracheostomy practice in patients with tetanus follows hospital policy of primary tracheostomy for airway control at both institutions receiving tetanus patients [24]. In other conditions tracheostomy was usually employed following 7–10 days of endotracheal intubation, following local protocols independent of the trial allocation.

### Endpoints

The primary endpoint was the proportion of patients with at least 1 episode of VARI during ICU stay. VARI was chosen in preference to VAP as it better reflects antibiotic use in this setting, and both are considered to have a common etiology. Furthermore, this definition does not include strict ventilator criteria which may be insensitive in LMIC settings where safety concerns are often the main determinant of ventilator settings. To explore the possibility that the intervention may delay VARI, we also assessed the time to develop VARI. The primary endpoint was evaluated by an ICU physician with access to all clinical data blinded to treatment allocation.

The core criteria for VARI required patients to have been intubated for at least 48 hours, the endotracheal tube to have been in situ within the 48 hours prior to onset and new antimicrobials to have been initiated to treat a new infection [23]. VARI was further defined as VAP if there were new or progressive changes on chest radiography and at least 2 of the following criteria: (1) axillary temperature >38°C or <36°C, (2) white blood cell count <4.0 10^9^/L or ≥12 × 10^9^/L with no other recognized cause, and (3) new onset of purulent respiratory secretions or change in character of sputum or increase in volume of sputum. VARI was defined as VAT if there was no new and persistent infiltrate on chest radiography and patients had criterion 3 plus either criterion 1 or 2.

Secondary endpoints included microbiologically confirmed VARI and VAP (based on the criteria for VAP/VAT above, plus growth of ≥10^5^ colony forming units/mL or equivalent semi-quantitative growth from endotracheal aspirates), mechanical ventilation/intubation duration, intubated days during which systemic antimicrobials were administered, incidence of other HAIs, length and cost of ICU/hospital stay, antimicrobials cost during ICU/hospital stay, and mortality at 28 and 90 days. Cost of antibiotics or ICU/hospital stay were derived from the bills presented to the patients or relatives for payment by them or their health insurance. This did not include staff costs. Safety endpoints included adverse events (defined according to Common Terminology Criteria for Adverse Events (CTCAE) v4.0 [25]).

### Sample Size and Randomization

Participating ICUs admit significant numbers of patients with tetanus, and these patients reflect a different population than other ICU patients, with longer duration of mechanical ventilation and less organ dysfunction (we have previously shown a lower incidence density of VARI among these [26]). Therefore, to preserve the generalizability of the study for settings where tetanus is less common, although still demonstrating the utility of the intervention in our population, we stratified the study population at a ratio of 3:7 by the admission diagnosis of tetanus and nontetanus.

Using data derived from a point prevalence study in Vietnam [27], we estimated the prevalence of VARI in nontetanus and tetanus ventilated patients was 20% and 30%, respectively. We calculated the sample size to detect a 40% reduction of VARI period prevalence (first occurrence during ICU stay) with 80% power and 5% significance (2-sided test), with a combined loss to follow-up and extubation within 48 hours (ie, before a diagnosis of VARI was possible) of 8%, to be 600 patients: 420 without and 180 with tetanus [23]. All patients were randomly assigned in a 1:1 ratio, stratified by site and tetanus diagnosis, to the control or intervention group using computer-generated block randomization.

### Statistical Analysis

Data were double entered into a database (CliRES) and analyzed using R (version 3.6.2, R Foundation for Statistical Computing, Vienna). The primary endpoint was analyzed in intention-to-treat (ITT) and per-protocol populations. For the primary endpoint in ITT population, patients who were intubated for <48 hours were considered not to have reached the primary endpoint. The primary endpoint (proportion with at least 1 episode of VARI) was analyzed using a logistic regression model with the randomized arm as main covariate and adjustment for tetanus status as main effect. Potential heterogeneity of the intervention effect was assessed on the basis of interaction tests and predefined subgroups, including patients with and without tetanus, patients with and without tracheostomy and hospital sites. For the time to develop VARI, we nonparametrically estimated cumulative incidence of VARI by tetanus status, with death/extubation/ICU discharge as competing risks. Curves were compared by log-rank test on subdistribution hazard and difference between arms was quantified via cause-specific proportional hazards model (standard Cox model) with adjustment for tetanus status as main effect. The statistical analysis plan was completed prior to unblinding (Supplementary Material). Following interim safety analysis, a specific analysis with for blood transfusion was included in this plan. All secondary outcomes were adjusted for tetanus status as main effect. Binary secondary endpoints (clinically and microbiologically confirmed VAP/HAI) were analyzed via logistic regression models. The distribution of duration of ventilation, intubation and ICU stay were estimated nonparametrically; death was considered a competing risk. The proportion of intubated days free of antimicrobials was analyzed using a Poisson regression model with number of intubated days without antimicrobials as outcome, the randomized arm as main covariate, and total (log-transformed) number of intubated days as offset. Quasi-likelihood was used to account for potential overdispersion. Direct hospital and ICU costs were taken from hospital bills and converted to USD as detailed in the statistical analysis plan. Hospital bills contained detailed costs of all drugs, procedures, treatments, and bed costs during patients’ stay. For the cost outcomes, we determined the best transformation via the Box-Cox procedure. After transformation, arms were compared using linear regression. We used a logistic regression model to quantify the probability of cuff pressure measurements within the target range for both interventions (post hoc analysis). We allowed the probability to change over time, by incorporating a linear trend on the logit scale. For quantification of uncertainty due to sampling variation, we used a bootstrap procedure (ClusterBootstrap) [28] that takes into account that individuals can have multiple cuff pressure measurements over time. Mortality was compared using Kaplan-Meier curves and modeled by Cox proportional hazards regression. The proportion of patients with at least one adverse event was compared using χ ^2^ test for independence, or Fisher exact test, if the expected number under the null hypothesis was ≤1 in ≥1 cells.

## RESULTS

**Figure 1.**
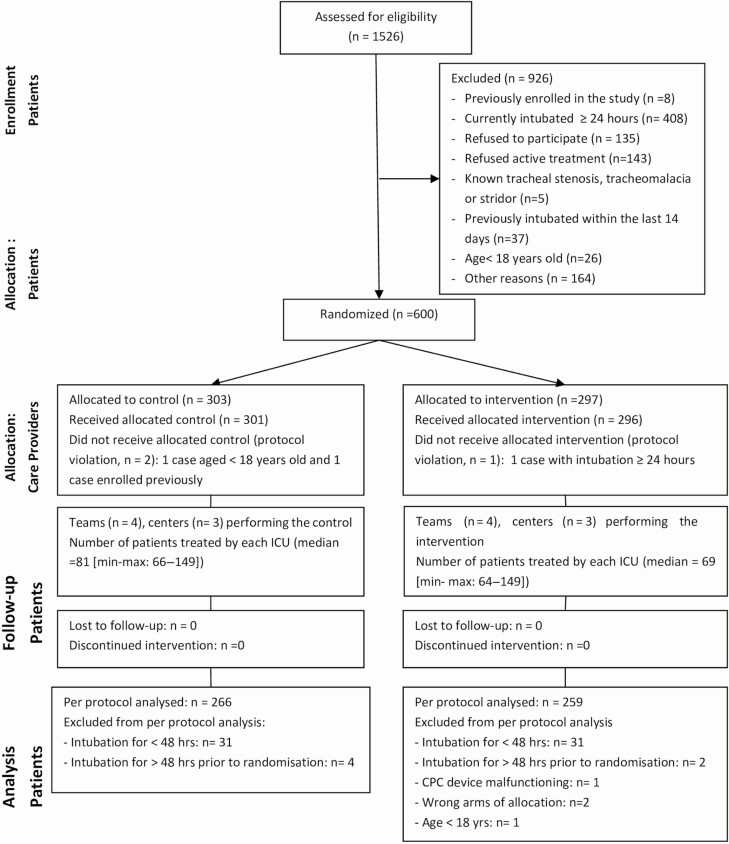
CONSORT flowchart of participants into the trial. Other reasons including: presumed to die within 48 hours after admission (39), hold on trial recruitment period (27), expected to extubation within 48 hours (13), transferred to another hospital within 24 hours (7), unable to provide the consent (5), and unspecified reasons (73). Abbreviations: CPC, cuff pressure control; ICU, intensive care unit.

Between November 2016 and December 2018, we screened 1526 patients and randomized 303 patients to receive intermittent CPC and 297 to receive continuous CPC. Three patients did not receive the allocated intervention (2 intermittent CPC and 1 continuous CPC) and had no data collected; these patients were not included in the analysis (Figure 1). The last follow-up was completed in March 2019. Sixty-nine percent (69.2%, 413/597) of patients were male and 25.6% (153/597) were initially ventilated by tracheostomy (98% of whom had tetanus). Baseline characteristics are summarized in Table 1. The median cuff pressures documented in the intermittent and continuous CPC arms were 20 cmH_2_O (IQR 15–22) and 25 cmH_2_O (IQR 25–25), respectively. The probability of cuff pressure measurements falling within the target range showed superiority of the continuous CPC device in maintaining pressures within the target range (Supplementary Figure 1). The rates of unplanned extubation and reintubation were similar between 2 arms (Supplementary Table 1).

**Table 1.** Baseline Demographics and Clinical Characteristics in Participants

Characteristic	Intermittent Cuff Pressure Control (N = 301)	Continuous Cuff Pressure Control (N = 296)
Age, years, median (IQR)	57 (43, 70)	58 (40, 70)
Sex		
Female	85/301 (28%)	99/296 (33%)
Male	216/301 (72%)	197/296 (67%)
Study site		
Trung Vuong hospital (TVH)	69/301 (23%)	66/296 (22%)
Hospital for Tropical Diseases (HTD)	148/301 (49%)	149/296 (50%)
National Hospital for Tropical Diseases (NHTD)	84/301 (28%)	81/296 (27%)
Transferred from other hospital	215/301 (71%)	211/296 (71%)
Time from intubation to randomization [hours]	12 (4, 19)	12 (5, 18)
Initially tracheostomy	79/301 (26%)	74/296 (25%)
Charlson score		
0	173/301 (57%)	191/296 (65%)
1–2	55/301 (18%)	27/296 (9%)
3–4	48/301 (16%)	47/296 (16%)
≥5	25/301 (8%)	31/296 (10%)
APACHE II, median (IQR)	17 (13, 21)	17 (13, 22)
Causes of admission		
Tetanus	91/301 (30%)	89/296 (30%)
Pneumonia (any)	87/301 (29%)	84/296 (28%)
Sepsis/septic shock	70/301 (23%)	75/296 (25%)
CNS infection	68/301 (23%)	61/296 (21%)
COPD	7/301 (2%)	3/296 (1%)
CVA	4/301 (1%)	5/296 (2%)
Myocardial infarction	15/301 (5%)	12/296 (4%)
Other causes^a^	20/301 (7%)	29/296 (10%)

Abbreviations: APACHE: Acute Physiology and Chronic Health Evaluation; CNS, central nervous system; COPD, chronic obstructive pulmonary disease; CVA, cerebrovascular accident; IQR, interquartile range.

^a^Other causes include: acute kidney injury, cholecystitis, coma, dengue, encephalitis, liver disease, malaria, pleural disease, poisoning, pulmonary edema, seizure, stroke, trauma, viral infection, other infection.

### Primary Outcome

**Figure 2.**
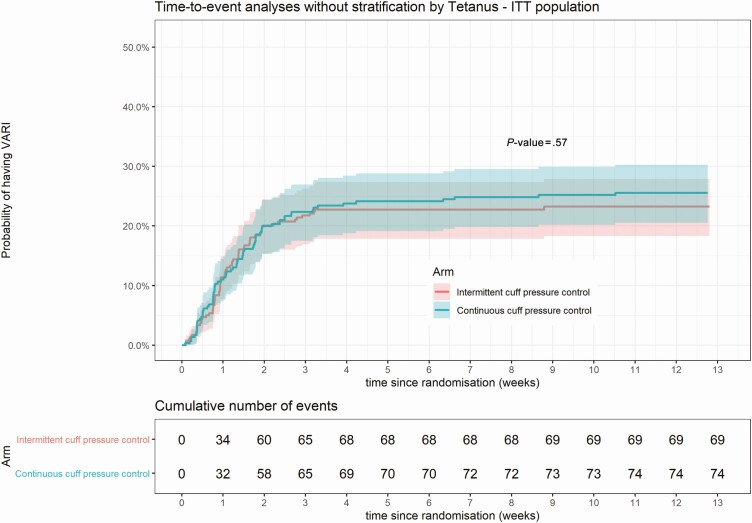
Time-to-event analyses without stratification by Tetanus—ITT population. Abbreviations: ITT, intention-to-treat; VARI, ventilator-associated respiratory infection.

At least 1 episode of VARI was observed in 24.0% (143/597) of patients. The per-protocol population included 87.9% (525/597) of patients, and VARI occurred in 27.8% (72/259) of patients with continuous CPC and 25.9% (69/266) with intermittent control. The proportion with at least 1 episode of VARI was similar in continuous and intermittent CPC arms in both intention-to-treat (OR 1.13; 95% CI .77–1.67) and per-protocol populations (OR 1.11; 95% CI .75–1.65). There was no difference in cause-specific event rate between the 2 arms in either population. (Table 2, Figure 2)

**Table 2.** Primary and Secondary Outcomes Among the Study Participant

	Intermittent Cuff Pressure Control (N = 301)		Continuous Cuff Pressure Control (N = 296)		Comparison	
	n	Summary Statistic	N	Summary Statistic	Effect Measure	P value
Primary outcome—ITT						
At least 1 episode VARI	301	69 (23%)	296	74 (25%)	OR 1.13 [0.77, 1.67]	.53
Cause-specific hazard [events/follow-up days]	301	69/3572	296	74/3929	HR 1.01 [0.73, 1.41]	.94
Primary outcome—per protocol						
At least 1 episode VARI	266	69 (26%)	259	72 (28%)	OR 1.11 [0.75, 1.65]	.60
Cause-specific hazard [events/follow-up days]	266	69/3540	259	72/3788	HR 1.02 [0.73, 1.41]	.93
Secondary outcomes						
Microbiologically confirmed VARI	301	57 (19%)	296	72 (24%)	OR 1.40 [0.94, 2.10]	.10
VAP	301	52 (17%)	296	51 (17%)	OR 1.00 [0.64, 1.55]	1.00
Microbiologically confirmed VAP	301	41/301 (14%)	296	50/296 (17%)	OR 1.32 [0.83, 2.10]	.24
Any HAI	301	108/301 (36%)	296	116/296 (39%)	OR 1.17 [0.83, 1.67]	.37
Proportion of antimicrobial-free intubated days	301	0.11 (0, 0.48)	296	0.1 (0, 0.43)	RP 0.99 [0.87, 1.12]	.89
Cause-specific hazard ICU discharge [events/follow-up days]	301	196/5785	296	194/5950	HR 0.95 [0.78, 1.16]	.6
Cause-specific hazard stopping ventilation [events/follow-up days]	301	283/4565	296	277/4815	HR 1.04 [0.55, 1.99]	.9
Cost of ICU stay (USD)	301	2231 (1317, 4430)	296	2427 (1438, 4159)	DTM 0.02 [−0.05, 0.08]	.62
Cost of ICU antimicrobials (USD)	301	243 (86, 701)	296	254 (90, 660)	DTM 0.02 [−0.25, 0.28]	.89
Cost of hospital stay (USD)	301	2425 (1449, 4627)	296	2754 (1694, 4496)	DTM 0.02 [−0.04, 0.08]	.45
ICU mortality risk	301	99/301 (33%)	296	95/296 (32%)	OR 0.96 [0.67, 1.38]	.81
28 day death probability (%) (tetanus +)	301	8.88 [2.81, 14.57]	296	7.95 [2.12, 13.44]	DM −0.92 [−9.07, 7.23]	.82
28 day death probability (%) (tetanus −)	301	40.0 [32.9, 46.3]	296	40.5 [33.3, 46.9]	DM0.49 [−9.02, 9.99]	.92
90 day death probability (%) (tetanus +)	301	10.1 [3.6, 16.1]	296	9.12 [2.89, 14.95]	DM −0.93 [−9.59, 7.74]	.83
90 day death probability (%) (tetanus −)	301	48.8 [41.5, 55.2]	296	52.3 [44.8, 58.7]	DM 3.44 [−6.29, 13.17]	.49

Abbreviations: DM, difference in mortality risk; DTM, difference in transformed means; HAI, hospital acquired infection, HR, hazard ratio, ICU, intensive care unit, OR, odds ratio; RP, relative proportion, VAP, ventilator-associated pneumonia; VARI, ventilator-associated respiratory infection.

### Secondary Outcomes

There were no significant differences between continuous and intermittent CPC with respect to all secondary endpoints (Table 2). Specifically, there was no difference in the proportion of antimicrobial-free intubated days or antimicrobial costs. Among 129 patients with VARI episodes, a diagnosis of VAP was made in 79.8% (103/129) patients, 88.3% (91/103) of whom were microbiologically confirmed (Supplementary Tables 2 and 3). The cause-specific rate of ICU discharge and stopping of ventilation were similar in both arms when stratified by tetanus diagnosis. The overall ICU mortality was 32.5% (194/597) and was not different between groups.

### Prespecified Subgroup Analysis of the Primary Endpoint

In the analysis by tetanus status, the proportion of patients with at least one episode of VARI was similar in patients with and without tetanus (OR 1.13; 95% CI .62–2.08 and OR 1.10; 95% CI .65–1.86, respectively) and cause specific hazards were also similar. There was no significant difference between the 2 arms in any other prespecified subgroups including by site or route of intubation (endotracheal or tracheostomy) (Table 3).

**Table 3.** Subgroup Analysis for Primary Endpoint (Intention-to-Treat Population)

	Intermittent Cuff Pressure Control (N = 301)		Continuous Cuff Pressure Control (N = 296)		Comparison	
Group	n	Summary Statistics	n	Summary Statistics	Effect Measure (95% CI)	P value
By tetanus status, OR						.86^a^
Tetanus −	210	34/210 (16%)	207	38/207 (18%)	OR 1.16 [0.70, 1.94]	.56
Tetanus +	91	35/91 (38%)	89	36/89 (40%)	OR 1.09 [0.60, 1.98]	.78
By tetanus status, cause-specific hazard [events/follow-up days]						.58^a^
Tetanus −	210	34/2309	207	38/2335	HR 1.1 [0.7, 1.8]	.66
Tetanus +	91	35/1264	89	36/1595	HR 0.92 [0.58, 1.47]	.74
By duration of intubation before randomization, OR^b^						.99^a^
<2 hours	36	13/36 (36%)	35	15/35 (43%)	OR 1.13 [0.42, 3.08]	.80
>2 hours	265	56/265 (21%)	261	59/261 (23%)	OR 1.13 [0.74, 1.72]	.58
By route of intubation as time-varying variable, cause-specific HR[events/follow-up days]^b^						.6^a^
Endotracheal tube	230	19/1763	225	25/1624	HR 1.39 [0.76, 2.57]	.29
Tracheostomy	141	48/1606	144	47/1785	HR 0.96 [0.63, 1.45]	.83
By site, OR^b^						.51^a^
TVH	69	5/69 (7%)	66	6/66 (9%)	OR 1.28 [0.37, 4.65]	.70
HTD	148	44/148 (30%)	149	42/149 (28%)	OR 0.93 [0.56, 1.56]	.79
NHTD	84	20/84 (24%)	81	26/81 (32%)	OR 1.54 [0.77, 3.12]	.22

Abbreviations: CI, confidence interval; HR, hazard ratio; HTD, Hospital for Tropical Diseases; NHTD, National Hospital for Tropical Diseases; OR, odds ratio; TVH, Trung Vuong hospital.

^a^Test for heterogeneity between subgroup and intervention arm.

^b^Adjusted for tetanus status as main effect.

### Safety Analysis

There was no difference in grade 3 and 4 adverse events between groups nor of tracheal complications, including bleeding (Table 4). The overall proportion of patients who received any blood products (packed red blood cells, fresh frozen plasma, or platelets) was 34.0% (100/294) in the continuous CPC arm and 24.1% (72/299) in the intermittent arm. Given this finding, further exploratory analyses were performed. There was no significant difference in receipt of any blood product for those with tetanus (OR 0.78; 95% CI .33–1.8). Patients without tetanus randomised to continuous CPC were significantly more likely to receive blood products (OR 1.98; 95% CI 1.32–3.0) and more often received red blood cells (RBCs) (39.6%, 82/207) or platelets (17.4%, 36/207) than those in the intermittent CPC arm (27.1% (57/210) for red blood cells, 10.0% (21/210) for platelets, *P* = .007 and .028, respectively). However, there was no difference between arms in the total volume of RBCs transfused, or the proportion of patients receiving cryoprecipitate or plasma regardless of tetanus status. When stratified by study site, significantly higher proportion of participants receiving blood products in the continuous CPC arm was observed in 2/3 centers (NHTD and TVH). Bleeding events were reported as grade 3/4 adverse events in 43 patients.

**Table 4.** Safety Reports—Cases With at Least One Episode of Specified Events

Event	Intermittent Cuff Pressure Control (N = 299)^a^	Continuous Cuff Pressure Control (N = 294)^a^	OR (95% CI)	P value
Any Grade 3/4 AEs	169 (56.3%)	184 (62.6%)	1.29 [.93, 1.79]	.16
Specified Grade 3 or 4 events or interventions				
ECMO	3 (1%)	1 (0.34%)	0.34 [.02, 2.65]	.63
Hypotension requiring vasopressor and/or inotropes	139 (46.5%)	135 (45.9%)	0.98 [.71, 1.35]	.95
Renal failure or renal replacement therapy	49 (16.4%)	64 (21.8%)	1.42 [.94, 2.15]	.12
Disseminated intravascular coagulation	22 (7.4%)	25 (8.5%)	1.17 [.64, 2.14]	.72
Blood product transfusion	72 (24.1%)	100 (34.0%)	1.63 [1.14, 2.33]	.01
Other Grade 3/4 AEs	47 (15.7%)	44 (15.0%)	0.94 [.60, 1.48]	.89
Tracheal related adverse Events				
Tracheal complications^c^	15 (5.0%)	22 (7.5%)	1.53 [.78, 3.07]	.28
Trachea related complications at 28 days ^b^	3/293 (1%)	3/286 (1.0%)		
Trachea related complications at 90 days^b^	3/207 (1.4%)	2/198 (1%)		

Abbreviations: AE, adverse event; CI, confidence interval; ECMO, extracorporeal membrane oxygenation; OR, odds ratio; SAE, serious adverse event.

^a^Data from 4 patients are missing from the safety analysis owing to leaving the study within 24 hours of enrolment (2 discharged home for palliation, 1 transferred to another hospital, 1 withdrew from the study).

^b^Not all patients could be contacted for 28 and 90 day follow-up.

^c^Tracheal complications: tracheal bleeding, tracheomalacia, tracheal stenosis, tracheooesophageal fistula.

## DISCUSSION

We report the largest pragmatic clinical trial to date evaluating the effectiveness of continuous CPC in preventing VARI. We found that continuous CPC did not result in a reduction or delay in VARI compared with intermittent CPC. Furthermore, we did not observe a difference in mortality, time on a ventilator, time in ICU, and antimicrobial use or costs nor did we see any difference in VAP, a more commonly used endpoint in other studies. Subgroup analysis did not identify groups where there was a benefit from the intervention. No adjustment was made for multiplicity of testing so results of secondary, safety, and exploratory analyses should be interpreted with this caveat.

Our intervention appeared effective in producing CPC consistently within the target range in the continuous group compared to the intermittent group and overall period prevalence for VAP (17%) was similar to results of a meta-analysis for lower middle-income (13.3%) and upper middle-income countries (15.4%), with similar microbiology [10].

Reasons for differences in our results compared to previous studies may lie in differences between study designs and contexts. Our study was designed as a pragmatic study in LMIC ICUs and therefore aimed to evaluate the intervention against the standard of care. As a result, we did not fully standardize infection control procedures, VARI prevention bundles, clinical management or equipment across sites. Spontaneous breathing trials are not routine in our hospital sites and furthermore are contraindicated in those with tetanus. Although head of bed elevation is routine in our study sites, a formal randomized controlled trial at our sites failed to show efficacy in VAP prevention [12]. Subgroup analysis by site did not show significant differences in primary outcomes and our study design should reduce differences between intervention groups, however it remains possible that allocations may have influenced routine practice.

Of potential importance, we used locally manufactured endotracheal tubes, which may be subject to structural irregularities and reliability issues not encountered in previous studies [19–21, 29] as tracheal sealing quality may vary by brand, size, and material [30]. This may reflect genuine product quality issues or may reflect changes in pressure within the system occurring as a result of connecting a hand-held manometer [31]. It is also possible that our choice of CPC device was important, as it has been suggested that rapid pressure correction with electronic versus pneumatic control devices may interfere with self-sealing processes [29, 32].

Adverse event analysis showed that the risk of anticipated adverse events associated with continuous and intermittent CPC was similar. Surprisingly, however, the proportion of patients on continuous CPC receiving blood products was higher. The significance of this finding is uncertain; it appeared to be restricted to participants without tetanus, in 2 of the three sites and was not associated with increased bleeding events, whereas both arms received similar volumes of red cells. In an individual patient data meta-analysis of 3 previous trials, the rate of red blood cell transfusion was not significantly different between continuous CPC (29% or 73/263) and intermittent CPC (26% or 73/280) (*P* = .359) [18].

In terms of trial design our study has addressed limitations of previous RCTs: the heterogeneity among small sample size trials using different devices and the unblinded assessment of VAP. We believe that consistent results across multiple study sites indicates reliability of our results. However, it is also possible that our study context is in itself a reason for discrepancy, with a different case-mix, particularly a high rate of tracheostomy at enrolment as a consequence of tetanus diagnosis. We believe that our choice of endpoints is a strength of this study. We used a blinded endpoint assessor and also collected data on other HAI and antibiotic use, enabling us to address concerns that in an open-label study, infection events may be classified and treated as nonrespiratory HAI.

## CONCLUSION

In conclusion, our study showed that compared to current standard of care, a continuous CPC device was not effective at preventing VARI in an LMIC setting.

## Supplementary Data

Supplementary materials are available at *Clinical Infectious Diseases* online. Consisting of data provided by the authors to benefit the reader, the posted materials are not copyedited and are the sole responsibility of the authors, so questions or comments should be addressed to the corresponding author.

ciab724_suppl_Supplementary_Material

ciab724_suppl_Supplementary_Tables_and_Figure

## Notes

***Author contributors.*** B. N., G. E. T., and D. W. developed the study conception. B. N., C. L. T., H. R. v. D., G. E. T., D. W., V. Q. D., L. M. Y., H. T. L., R. B. G., D. H. K. T., H. B. L., J. C., E. K., N. V. H., L. T. C. designed the study. V. Q. D., L. M. Y., H. T. L., V. D. P., N. T. H. M., N. H. P., N. P. H. L., T. P. T., D. T. H., N. V. T., N. T. C., D. T. T., N. T. H., N. T. T. V., V. T. T. L., T. T. Q. N., H. B. L., N. T. T. H., N. T. T. V., J. C., E. A., N. V. H., L. T. C., C. L. T., and B. N. implemented the study. R. B. G. and D. H. K. T. performed statistical analysis. V. Q. D., B. N., C. L. T., H. R. v. D., G. E. T., R. B. G., D. H. K. T., E. A., and D. W. performed data interpretation. V. Q. D., B. N., H. R. v. D., R. B. G., C. L. T., and G. E. T. drafted the manuscript. All authors critically reviewed and approved the final manuscript.

***Acknowledgments.*** The authors thank all patients who participated in this study for their time and voluntary involvement. They thank all staff in ICUs, laboratory, and radiology departments in the National Hospital of Tropical Diseases, Hospital for Tropical Diseases and Trung Vuong Emergency Hospital for their great contribution to the study.

***Financial support.*** The work was supported by the Wellcome Trust (grant number 106680/B/14/Z).

***Potential conflicts of interest.*** The authors: No reported conflicts of interest. All authors have submitted the ICMJE Form for Disclosure of Potential Conflicts of Interest. Conflicts that the editors consider relevant to the content of the manuscript have been disclosed.
