# Supplementary material for: Effectiveness of Continuous Endotracheal Cuff Pressure Control for the Prevention of Ventilator-Associated Respiratory Infections: An Open-Label Randomized, Controlled Trial
Source: Clin Infect Dis. 2021 Aug 22;74(10):1795–803. doi: 10.1093/cid/ciab724 (PMC9155610; doi:10.1093/cid/ciab724)
Supplement: ciab724_suppl_Supplementary_Material [file ciab724_suppl_supplementary_material.docx]

# Statistical Analysis Plan for 16HN Continuous versus intermittent endotracheal cuff pressure control for the prevention of ventilator-associated respiratory infections in Vietnam. NCT02966392

| Version Number & date: 16HN SAP 1.0 20JAN2020 | | | |
| --- | --- | --- | --- |
| **Author** | **Position** | **Signature** | **Date** |
| Ronald Geskus | Trial Statistician | 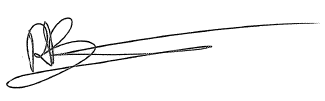 | Jan 20, 2020 |
| Behzad Nadjm | OUCRU PI |  |  |

**Revision History**

| **Version** | **Author** | **Date** | **Reason for Revision** |
| --- | --- | --- | --- |
|  |  |  |  |
|  |  |  |  |
|  |  |  |  |

Table of Contents

[Statistical Analysis Plan for 16HN Continuous versus intermittent endotracheal cuff pressure control for the prevention of ventilator-associated respiratory infections in Vietnam. NCT02966392 1](#_Toc29994985)

[Purpose of this document 3](#_Toc29994986)

[Statistical software 3](#_Toc29994987)

[Some recoding of variables: 3](#_Toc29994988)

[Selection of the analysis populations 3](#_Toc29994989)

[Intention to treat analysis 3](#_Toc29994990)

[Per protocol analysis: 4](#_Toc29994991)

[Defining patients not eligible for the per protocol analysis: 4](#_Toc29994992)

[Derivation of Table 1 6](#_Toc29994993)

[Age 6](#_Toc29994994)

[Male sex 6](#_Toc29994995)

[Study Site 6](#_Toc29994996)

[Transfer from other hospital 6](#_Toc29994997)

[Time from intubation to randomization 6](#_Toc29994998)

[Initially tracheostomy 6](#_Toc29994999)

[Charlson score 7](#_Toc29995000)

[APACHEII 8](#_Toc29995001)

[Cause of admission 11](#_Toc29995002)

[Derivation of Table 2 and Table 3. 13](#_Toc29995003)

[Primary Outcome 13](#_Toc29995004)

[At least one episode of VARI (adjusted for whether they have tetanus) 13](#_Toc29995005)

[Secondary Outcomes 14](#_Toc29995006)

[Microbiologically confirmed VARI 14](#_Toc29995007)

[VAP 15](#_Toc29995008)

[Microbiologically confirmed VAP 15](#_Toc29995009)

[Any HAI 15](#_Toc29995010)

[Proportion of intubated days without antibiotics (after randomization) 15](#_Toc29995011)

[ICU stay 17](#_Toc29995012)

[Ventilated Days 17](#_Toc29995013)

[Cost of ICU Stay 18](#_Toc29995014)

[Cost of ICU antibiotics 18](#_Toc29995015)

[Cost of hospital stay 19](#_Toc29995016)

[ICU Mortality 19](#_Toc29995017)

[Overall mortality; 28 and 90 Day mortality. 19](#_Toc29995018)

[Derivation of Table 3 21](#_Toc29995019)

[Tetanus 21](#_Toc29995020)

[Intubation <=2 hours before randomization 21](#_Toc29995021)

[ET Tube/Trache: 21](#_Toc29995022)

[Site 21](#_Toc29995023)

[Derivation of Table 4 22](#_Toc29995024)

[Total Grade 3/4 AEs 23](#_Toc29995025)

[Number with at least one Grade 3/4 AE 23](#_Toc29995026)

## Purpose of this document

Describes the planned analyses and endpoint derivations for the above trial as outlined in the study protocol (16HN PROTOCOL VARI prevent EN V2.0 03MAR17) and trial protocol paper (Dat *et al* Trials 2018 19:217). Where this analysis plan differs from that in either of these documents, this has been highlighted and explained.

It focuses on the analysis for the main clinical trial publication and does not include analysis for any subsidiary studies.

*Derivation for identification of relevant data points for statistical analysis will be given in italics using variable names from the CLiRES database.*

Where a date is given with no time, the time will be considered as midday (12:00Hrs)

Variables generated for the description of statistical analysis (ie not directly copied from CLIRES) will be given in Red

Except where otherwise noted, records with missing data will be excluded and a description of the number of excluded participants/episodes described.

Statistical adjustment for multiplicity testing will not be deployed, rather interpretation of results will consider this issue.

## Statistical software

All statistical analyses will be performed with the statistical software R using the current R version at the time of the final analysis (R Foundation for Statistical Computing, Vienna, Austria). The versions of R and R packages used are given in the report.

## Some recoding of variables:

To ensure a single date for hospital discharge and ICU discharge for those discharged directly from ICU

IF *FINISH.ICUDate* = *FINISH.HosDate* THEN RECODE *FINISH.HosSame* = True

IF *FINISH.HosSame* = True THEN RECODE *FINISH.HosDate = FINISH.ICUDate*

## Selection of the analysis populations

The number of patients excluded from this analysis, the reason for exclusion and their allocated arm will be listed in the CONSORT diagram*.*

### Intention to treat analysis

This will include all randomized patients who received the intervention or control, other than patients who withdrew consent and did not want their data used at all. Patients who withdrew consent or were withdrawn by medical staff but permitted analysis of their data up to that point in time are included until the time point at which they withdrew consent/were withdrawn.

*FINISH.IsCompleted = 2 (which means “No, withdrew consent”) then we can only use the data if they permitted use of data up to this point, defined by FINISH.IsAgree = Yes.*

*(ie exclude completely only if FINISH.IsAgree = No and FINISH.IsCompleted = No, withdrew consent).*

Other study codes that are not associated with patients that received the treatment will be identified through analysis of data in protocol deviation/violation logs and notes-to-file based on records of study numbers included in the randomization list but not found elsewhere in the database.

### Per protocol analysis:

Based on the ITT population, this will **additionally** **exclude** patients who **did not** meet the inclusion criteria and/or met the exclusion criteria below **and those who were intubated for less than 48 hours after randomization,** including those who withdrew consent within 48 hours of randomization (as these patients could not meet the primary endpoint).

Inclusion criteria: Aged ≥ 18 years

About to be intubated or intubated for ≤ 24 hours

(either oral or tracheostomy)

For active treatment (ie physician caring for patient would prescribe an antibiotic if the patient developed an infection)

Exclusion criteria: Previously enrolled in the study

Previous completed episode of intubation in the last 14 days

Known tracheal stenosis, tracheomalacia or stridor.

Lack of informed consent.

The intention to treat population will be used for all the listed analyses, the per protocol analysis will be performed on the primary endpoint (proportion of patients in each arm with at least one episode of VARI, the cause-specific cumulative incidence and the cause-specific hazard for VARI) in all patients and in the tetanus and non-tetanus subgroups. If the intention to treat and per-protocol analyses on the primary endpoint lead to inconsistent results, then per-protocol analysis will also be carried out on all the other endpoints. The decision of what constitutes inconsistency will be made after un-blinding and the primary endpoint analysis.

Patients who were recruited despite violating other enrolment criteria will be identified through analysis of data in protocol deviation/violation logs and notes-to-file.

### Defining patients not eligible for the per protocol analysis:

- Age <18 years

Age is described by ENR.Age OR (ENR.RandDate – ENR.YOB) if ENR.Age is missing, assume 1^st^ January as birth date for YOB

- Intubated for more than 24 hours prior to randomization.

*[ENR.RandDate + ENR. RandTime] – [ADM.Date1stIntu +ADM.Time1stIntu]* > 24 hours

- Intubated for <48 hours

To determine the duration each participant was intubated before leaving the study (described by a new variable we must generate called DurIntub):

*ADM.Date1stIntu + ADM.Time1stIntu* determines the start time.

The end time of intubation (described by a new variable we must generate called EndIntub) is the earliest of :

1. 90 days after randomization.(*ENR.RandDate +90* days)
2. *FINISH.ICUDate* (Midday of Date of ICU discharge/death)
3. *FINISH.CompleteDate* (Midday of Date of withdrawl from the study.

Note that if *FINISH.IsCompleted* = 2 (“No, withdrew consent”) then we can only use the data if they permitted use of data up to this point, defined by *FINISH.IsAgree* = Yes. For other causes of *FINISH.IsCompleted* = No (values 3 or 4)we can use the data

1. Extubation Time: This is established as the latest accidental or deliberate extubation, which is not followed by a reintubation event. Thus there may not be an extubation time if either there is no extubation event or every extubation is followed by a reintubation event. Reintubation events include tracheostomy and reinsertion of an ETT tube. If there is no Extubation time then the EndIntub is defined by *FINISH.ICUDate* or *FINISH.CompleteDate* if *FINISH.IsCompleted* =2 or 3 (withdrew) or 90 days after randomisation

So Extubation time it is the latest of:

[*AIR.AccidentDate* + *AIR.AccidentTime*]

or [*AIR.DeliberateDate* + *AIR.DeliberateTime*]

or *SUPAIR.SUPDate*+*SUPAIR.SUPTime* if *SUPAIR.EventName*= 1 (i.e. “Deliberate extubation”)*

or *SUPAIR.SUPDate*+*SUPAIR.SUPTime* if *SUPAIR.EventName*= 3 (i.e. “Accidental extubation”)*

which is not followed by one of the following

*AIR.TrachDate* (tracheostomy inserted after extubation)

*AIR.ReintuDate3* (reintubated after accidental extubation)

*AIR.ReintuDate4* (reintubated after deliberate extubation)

*SUPAIR.SUPDate*+*SUPAIR.SUPTime* if *SUPAIR.EventName*= 5 (i.e. “reintubation”)*

*please note that there may be more than one *SUPAIR.EventName*= Deliberate extubation, Accidental extubation, or  reintubation for one participant

Thus DurIntub = EndIntub *– (ADM.Date1stIntu + ADM.Time1stIntu)*

If DurIntub <48 hours they will be excluded from the per protocol analysis

**Table 1** – Baseline characteristics of enrolled patients

|  | Intervention  N = | Control  n = |
| --- | --- | --- |
| Age (median, 1st and 3^rd^ quartile) |  |  |
| Sex |  |  |
| Study Site |  |  |
| NHTD |  |  |
| HTD |  |  |
| BVTV |  |  |
| Transferred from other hospital |  |  |
| Time from intubation to randomization in hours (median 1st and 3^rd^ quartile) |  |  |
| Initially tracheostomy |  |  |
| Charlson score (median 1st and 3^rd^ quartile) |  |  |
| APACHE II (median 1st and 3^rd^ quartile) |  |  |
| Cause of Admission |  |  |
| Tetanus |  |  |
| Pneumonia (any) |  |  |
| Sepsis/septic shock |  |  |
| CNS Infection |  |  |
| COPD |  |  |
| CVA |  |  |
| MI |  |  |
| Other |  |  |

## Derivation of Table 1

Age**:** *ENR.Age OR (ENR.RandDate – ENR.YOB)* if *ENR.Age* is missing, assume 1^st^ January as birth date for *YOB*

Male sex**:** *ENR.Sex*

Study Site: derived from the first 3 numbers of the Patient Code

*020* = NHTD

*003* = HTD

*103* = BVTV

Transfer from other hospital**:** *ADM.source*

Time from intubation to randomization***:*** *[ ENR.RandDate-ENR.RandTime] - [ADM.Date1stIntu + ADM.Time1stIntu]*

Initially tracheostomy: *ADM.NKQ (1=Tracheostomy; 2=Mouth)*

Charlson score**:**

Sum all of the weights for the patient, weights shown below by CLIRES code

Where data are missing, consider as not present (ie NA= No)

| **Assigned weight for disease** | **Conditions** | **CLIRES code for 16HN** |
| --- | --- | --- |
| 1 | Myocardial infarct | *ADM.Myo* |
|  | Congestive heart failure | *ADM.CongI_III* |
|  | Peripheral vascular disease | *ADM.Peri* |
|  | Cerebrovascular disease | *ADM.Cere* |
|  | Dementia | *ADM.Deme* |
|  | Chronic pulmonary disease | *ADM.Chro* |
|  | Connective tissue disease | *ADM.Conn* |
|  | Ulcer disease | *ADM.Peptic* |
|  | Mild liver disease | *ADM.Mild* |
|  | Diabetes | *ADM.DiaCo* |
| 2 | Hemiplegia | *ADM.Hemi* |
|  | Moderate or severe renal disease | *ADM.ModeSeve* |
|  | Diabetes with end-organ damage | *ADM.DiaDa* |
|  | Any tumor, Leukemia, Lymphoma | *ADM.AnyMa** |
|  |  |  |
|  |  |  |
| 3 | Moderate or severe liver disease | *ADM.Mode* |
| 6 | Metastatic solid tumor | *ADM.Meta* |
|  | AIDS | *ADM.AIDS* |

*This counts only once ie if the CRF has *ADM.AnyMa*=TRUE they score 2 points for that

### APACHEII

The Apache-II Score provides an estimate of ICU mortality based on a number of laboratory values and patient signs taking both acute and chronic disease into account. Note: The data used should relate to the 24 hours after randomisation and the worst (highest scoring) recorded values should be used.

**The final score sums scores from table A (physiology), B (Age), C (Chronic health)**

**A: Physiological score.** This the sum of the scores obtained from the 12 parameters below, each patient scores from 0 to 4 for each of the first 11 parameters. GCS is scored from 0 to 12 (actual GCS runs from 3 to 15)

| APACHE Parameter | CLIRES CODE or derivation | SCORE | | | | | | | | |
| --- | --- | --- | --- | --- | --- | --- | --- | --- | --- | --- |
|  |  | +4 | +3 | +2 | +1 | 0 | +1 | +2 | +3 | +4 |
| Temperature | See below a | ≥ 41 | 39-40.9 |  | 38.5-38.9 | 36-38.4 | 34-35.9 | 32-33.9 | 30-31.9 | ≤ 29.9 |
| Mean Arterial BP (MAP) | See below b | ≥ 160 | 130-159 | 110-129 |  | 70-109 |  | 50-69 |  | ≤ 49 |
| Heart Rate | See below c | ≥ 180 | 140-179 | 110-139 |  | 70-109 |  | 55-69 | 40-54 | ≤ 39 |
| Respiratory Rate | See below d | ≥ 50 | 35-49 |  | 25-34 | 12-24 | 10-11 | 6-9 |  | ≤ 5 |
| A-aPO_2_ (If FiO_2_≥ 50%)  PaO_2_ (If FiO_2_ < 50%) | See below e | ≥ 500 | 350-499 | 200-349 |  | < 200  > 70 | 61-70 |  | 55-60 | < 55 |
| Arterial pH  ^1^Serum HCO_3_^-^ | See below f | ≥ 7.7  ≥ 52 | 7.6-7.69  41-51.9 |  | 7.5-7.59  32-40.9 | 7.33-7.49  23-31.9 |  | 7.25-7.32  18-21.9 | 7.15-7.24  15-17.9 | < 7.15  < 15 |
| Serum Na^+^ | *APAC.Sodium* | ≥ 180 | 160-179 | 155-159 | 150-154 | 130-149 |  | 120-129 | 111-119 | ≤ 110 |
| Serum K^+^ | *APAC.Potassium* | ≥ 7 | 6-6.9 |  | 5.5-5.9 | 3.5-5.4 | 3-3.4 | 2.5-2.9 |  | < 2.5 |
| Serum Creatinine | *APAC.Creatinine* | ≥ 3.5 | 2-3.4 | 1.5-1.9 |  | 0.6-1.4 |  | < 0.6 |  |  |
| Hematocrit | *APAC.Haematocrit* | ≥ 60 |  | 50-59.9 | 46-49.9 | 30-45.9 |  | 20-29.9 |  | < 20 |
| WBC Count | *APAC.BloodCell* | ≥ 40 |  | 20-39.9 | 15-19.9 | 3-14.9 |  | 1-2.9 |  | < 1 |
| GCS | See below g | Score = 15 – Actual GCS | | | | | | | | |
|  | | ^1^Use only if no ABGs | | | | | | | | |

**Unless otherwise stated below, if any value is missing it should be considered as normal (score of zero for that component)**

1. Temperature.

Maximum and minimum temperature are coded in *DAILY1n2.TempMax* and *DAILY1n2.TempMin*, where *DAILY1n2.VSDate*=*ENR.RandDate* (using the first DAILY entry). The value that derives the highest score from the chart above should be used.

1. Mean Arterial BP (MAP).

This is calculated from *APAC.Systolic* and *APAC.Diastolic* by the equation MAP = {(*APAC.Diastolic* *2) +*APAC.Systolic*}/3

1. Heart Rate

Max and minimum heart rate are given in *APAC.PuHightest* and *APAC.PuLowest* respectively. The value that derives the highest score from the chart above should be used.

1. Respiratory Rate

Max and minimum respiratory rate are given in *APAC.ResHightest* and *APAC.ResLowest*.

The value that derives the highest score from the chart above should be used.

1. A-aPO2 and PaO2

If *APAC.FiO2* <0.5 OR APAC.FiO2 is blank, then score depends on *APAC.PaO2*:

| *APAC.PaO2* | Score |
| --- | --- |
| >70 | 0 |
| 61-70 | + 1 |
|  | + 2 |
| 55-60 | + 3 |
| <55 | + 4 |

If *APAC.FiO2* ≥ 0.5 then calculate Aa Gradient, and use it in the table below to generate the Aa gradient and thus the score:

Aa gradient = (713 x *APAC.FiO2*) – (*APAC.PaCO2*/0.8) – *APAC.PaO2*

| Aa Gradient (generated) | Score |
| --- | --- |
| <200 | 0 |
|  | + 1 |
| 200 – 349 | + 2 |
| 350 – 499 | + 3 |
| ≥500 | + 4 |

Note that if *APAC.PaCO2* is missing it can be calculated from the following:

*APAC.HCO3*/((0.03*(10^ (*APAC.ArterialpH*-6.1)))

1. Arterial pH/Bicarbonate

Use arterial pH if it is available, this is given by *APAC.ArterialpH*

If *APAC.ArterialpH* is missing use *APAC.HCO3*

1. Glasgow Coma Score (GCS)

If *APAC.GlasgowScore* != Preintubation then use Actual GCS = 15 (so APACHE score for this variable is 15-15= 0)

If *APAC.GlasgowScore* = Preintubation then use the score as derived below,

If *APAC.Motor* AND *APAC.Verbal* AND *APAC.Eyes* all do not equal 0 and are not missing and are not 9, then actual GCS= *APAC.Motor* + *APAC.Verbal* + *APAC.Eyes*

If any of *APAC.Motor* AND *APAC.Verbal* AND *APAC.Eyes* is missing or equals 0 or 9 then actual GCS = *APAC.Total*

**B: Age score**

| Age (Yrs) | SCORE |
| --- | --- |
| ≤ 44 | 0 |
| 45-54 | 2 |
| 55-64 | 3 |
| 65-74 | 5 |
| ≥ 75 | 6 |
|  |  |

Age is given as above: *ENR.Age*

if *ENR.Age* is missing use *(ENR.RandDate – ENR.YOB*), assume 1^st^ January as birth date for YOB

**C: chronic health score**

If all of the following = False: *ADM.Mode; ADM.CongIV*; *ADM.Seve*; *ADM.AIDS*

Then the patient has no chronic health score (ie Score for C=0)

If any of these 4 variables (*ADM.Mode; ADM.CongIV*; *ADM.Seve*; *ADM.AIDS)* = True then the patient has a chronic health score, the value for C is determined according to *ADM..Elective* and *ADM.Emergency* values as follows:

If *ADM.Elective*=No or NA AND *ADM.Emergency*=No or NA

Then score for C=5

If *ADM.Emergency*=Yes, regardless of result for *ADM.Elective*

Then score for C=5

If *ADM.Elective*=Yes AND *ADM.Emergency*=No or NA

Then score for C=2

**The final APACHE II Score for Table 1 sums scores from table A (physiology), B (Age), C (Chronic health)**

Cause of admission *(note these are not mutually exclusive):*

Tetanus – *ADM.Tetanus*

Pneumonia - *ADM.Pneumonia*

Sepsis/septic shock *- ADM.Sepsis OR ADM.Septic*

CNS Infection *– ADM.CNS*

COPD *– ADM.COPD*

CVA *– ADM.CVA*

Myocardial Infarction – *ADM.Myo*

Other *–ADM.SpOther,* when *ADM.Other = True* (in some cases the text in *ADM.SpOther* has been deemed by investigators, blinded to the allocation and prior to the analysis, to be detail of an already specified cause of admission or to fall within the pre-specified categories above)

**Table 2 Primary and secondary outcomes**

|  | **Intervention**  **N=** | **Control**  **N=** | **effect measure**  **(95% CI)** | **p** |
| --- | --- | --- | --- | --- |
| **Primary Outcome - ITT** |  |  |  |  |
| At least one episode VARI | **events/N (%)** | **events/N (%)** | **OR** |  |
| Cause-specific hazard | **events/total follow-up days** | **events/total follow-up days** | **HR** |  |
| **Primary Outcome – per protocol** |  |  |  |  |
| At least one episode VARI | **events/N (%)** | **events/N (%)** | **OR** |  |
| Cause-specific hazard | **events/total follow-up days** | **events/total follow-up days** | **HR** |  |
| **Secondary Outcomes** |  |  |  |  |
| Microbiologically Confirmed VARI | **events/N (%)** | **events/N (%)** | **OR** |  |
| VAP | **events/N (%)** | **events/N (%)** | **OR** |  |
| Microbiologically confirmed VAP | **events/N (%)** | **events/N (%)** | **OR** |  |
| Any HAI | **events/N (%)** | **events/N (%)** | **OR** |  |
| Proportion of intubated days without antibiotics | **total days without antibiotics/total days intubated** | **total days without antibiotics/total days intubated** | **Relative proportion** |  |
| time to ICU discharge | **events/total follow-up days** | **events/total follow-up days** | **hazard ratio** |  |
| Ventilated days | **events/total follow-up days** | **events/total follow-up days** | **hazard ratio** |  |
| Cost of ICU stay | **median (1^st^-3^rd^ quartile)** | **median (1^st^-3^rd^ quartile)** | **transformed mean costs** |  |
| Cost of ICU antibiotics | **median (1^st^-3^rd^ quartile)** | **median (1^st^-3^rd^ quartile)** | **transformed mean costs** |  |
| Cost of Hospital stay | **median (1^st^-3^rd^ quartile)** | **median (1^st^-3^rd^ quartile)** | **transformed mean costs** |  |
| ICU Mortality Risk | **events/N (%)** | **events/N (%)** | **OR** |  |
| Mortality at 28 days  Patients with tetanus | **Estimate based on Kaplan-Meier (95% CI)** | **Estimate based on Kaplan-Meier (95% CI)** | **risk difference** |  |
| Mortality at 28 days  Patients without tetanus | **Estimate based on Kaplan-Meier (95% CI)** | **Estimate based on Kaplan-Meier (95% CI)** | **risk difference** |  |
| Mortality at 90 days  Patients with tetanus | **Estimate based on Kaplan-Meier (95% CI)** | **Estimate based on Kaplan-Meier (95% CI)** | **risk difference** |  |
| Mortality at 90 days  Patients without tetanus | **Estimate based on Kaplan-Meier (95% CI)** | **Estimate based on Kaplan-Meier (95% CI)** | **risk difference** |  |

Note that the N in each cell can be smaller than the N in the header because of missing values.

## Derivation of Table 2 and Table 3.

The primary outcome will be analysed using a logistic regression model with the randomised arm as the main covariate and adjustment for tetanus status as a main effect. Patients who are intubated for < 48 h will be regarded as not having reached the primary endpoint (for ITT). Potential heterogeneity of the intervention effect will be assessed on the basis of interaction tests and the predefined subgroups, including (1) patients with and without tetanus, (2) patients intubated for ≤ 2 and > 2 h before randomisation, (3) patients with and without tracheostomy and (4) hospital site (Table 3).

The primary outcome (at least one episode of either ventilator associated pneumonia (VAP) or ventilator associated tracheobronchitis(VAT)) was assessed by a senior intensive care doctor who was not involved in the management of the patients and was blinded to the allocated arm of the study. The assessor scrutinized the clinical details and microbiological results as recorded in the case record file together with the radiology or radiology reports and compared these with the pre-defined criteria for VAP and VAT. The same assessor determined the outcome for all patients. Thus when a patient had a defined clinical episode that could be VAP or VAT an HAISUM form was filled out. Each patient may then have from zero to X HAISUM forms filled. The different HAISUM forms for a patient are identified by the *HAISUM.HAINo* (which will run from 1 to X). Any one of these could have a result of *HAISUM.VAP* or *HAISUM.VAT*=True defining them as having had VAP or VAT respectively.

For the logistic regressions in Table 2 and Table 3, we use the likelihood ratio test. For all other analyses we use the Wald test or t-test.

Intervention/control is derived from the randomization list.

Tetanus status is derived from *ENR.IsTetanus*

### Primary Outcome

### At least one episode of VARI (adjusted for whether they have tetanus)

This is true when *HAISUM.VAP*=True OR *HAISUM.VAT*=True for any value of *HAISUM.HAINo* which corresponds to that participant (designated by *HAISUM.PatientNo*)

Please note – each participant (identified through *HAISUM.PatientNo*, can have more than one *HAISUM.VAP* or *HAISUM.VAT* result. These are discriminated by different (sequential) *HAISUM.HAINo* results. If any *HAISUM.VAP* or *HAISUM.VAT* = TRUE then the patient has had at least one episode of VARI.

If a patient withdraws, and consent to using their data up to that point then we would use their VARI status at withdrawal. If they withdrew and withdrew consent completely they will have been excluded from the ITT and PP analyses already.

After scrutiny of the previous literature and discussion of the possibility that the intervention may delay VARI we additionally estimate and plot the cumulative incidence of VARI by tetanus status, with death/extubation/ICU discharge as combined competing risk. For VARI, curves are compared by the log-rank test on the subdistribution hazard. We also fit a cause-specific proportional hazards model (i.c. a standard Cox model) with adjustment for tetanus status as main effect. For both the logistic regression model and the cause-specific hazards regression model, we test for heterogeneity of the intervention effect by tetanus status and report the hazard ratios (Table 3).

For this analysis,time of VARI is dictated by the earliest HAISUM.HAIEDate when *HAISUM.VAP*=True OR *HAISUM.VAT=True* for any value of *HAISUM.HAINo* which corresponds to that participant (designated by *HAISUM.PatientNo*)

Time of death/ICUdischarge/extubation is stipulated by *EndIntub* (page 5)

For patients that withdrew, they are censored at the time of withdrawal, this date is defined as *FINISH.CompleteDate* where *FINISH.Complete* = 2 or 3 (withdrew consent/withdrawn by doctor)

### Secondary Outcomes

All secondary outcomes will be adjusted for tetanus status as main effect (except for the nonparametric time-to-event curves, which are computed separately for patients with and without tetanus).

Binary secondary endpoints (clinical and microbiologically confirmed VAP and any HAI) will be analysed in the same way as the primary endpoint.

The distribution of duration of ventilation, intubation (i.e., time to extubation) and the time of ICU stay will be estimated; death will be considered as a competing event. We will nonparametrically estimate the cause-specific cumulative incidence for both event types and plot the results. Cause-specific cumulative incidence functions will be compared between the arms using a Fine and Gray regression model using tetanus status as a stratum variable. We will also fit a cause-specific proportional hazards model.

The proportion of intubated days free of antibiotics will be analysed using a Poisson regression model with the number of intubated days without antibiotics as the outcome, the randomized arm as the main covariate, and the (log-transformed) total number of intubated days as an offset. Quasi-likelihood will be used to account for potential over-dispersion.

The cost outcomes have a skewed distribution. Therefore we use the Box-Cox procedure with intervention arm and tetanus status as covariables to find a suitable transformation. If reasonable, we use the identity or log transformation. After the transformation, arms are compared using linear regression. As effect measure we report the difference in expected value on the transformed scale (and report the transformation we used). Since this may be hard to interpret, we also plot the distribution of the cost variable by intervention arm and tetanus status via histograms. We add to the histograms the mean value with 95% confidence interval for each combination of intervention arm and tetanus status.

**Mortality will be visualised in each arm using Kaplan-Meier curves and modelled using Cox regression.**

For all time-to-event analysis patients that withdrew will be censored at the time of withdrawal.

### Microbiologically confirmed VARI

This is true when *HAISUM.VAP*=True OR *HAISUM.VAT*=True for any value of *HAISUM.HAINo* which corresponds to that participant (designated by *HAISUM.PatientNo*)

AND

*HAISUM.MicroConfirm*=True for that episode (designated by *HAISUM.PatientNo* and *HAISUM.HAINo*)

### VAP

This is true when *HAISUM.VAP*=True for any value of *HAISUM.HAINo* which corresponds to that participant (designated by *HAISUM.PatientNo*)

### Microbiologically confirmed VAP

This is true when *HAISUM.VAP*=True for any value of *HAISUM.HAINo* which corresponds to that participant (designated by *HAISUM.PatientNo*)

AND

*HAISUM.MicroConfirm*=True for that episode (designated by *HAISUM.PatientNo* and *HAISUM.HAINo*)

### Any HAI

This is true when any of the below are true for any value of *HAISUM.HAINo* which corresponds to that participant (designated by *HAISUM.PatientNo*)

*HAISUM.VAP*=True

*HAISUM.VAT*=True

*HAISUM.HAIOTH*=True

### Proportion of intubated days without antibiotics (after randomization)

For each patient we need to define each day as an ET day, a Tracheostomy day or a not-intubated day. ET days and Tracheostomy days together define intubated days.

*Calculation of ET days*

An ET day is defined as a day between (inclusive of 1^st^ and last days)

Start:

IF *ADM.NKQ* = mouth: latest (farthest forward in time) of *ADM.Date1stIntu* OR *ENR.RandDate*

IF *ADM.NKQ* = tracheostomy, they are never an ET Patient (always Trache)

Stop date = earliest of *AIR.TrachDate* if *AIR.IsTrach* = Yes OR EndIntub (see Page 3 for derivation of EndIntub)

However some participants may have had their ET days interrupted.

We would only consider an intubation interrupted if the interruption lasts 2 full days (ie not including the day that the intubation stopped or the day that it restarted).

Patients with breaks in ET day can be seen by:

*AIR.Accident* = Yes AND *AIR.IsReintu3* = Yes

(indicating they were accidentally extubated and then reintubated)

OR

*AIR.Deliberate* = Yes AND *AIR.IsReintu4 = Yes*

Indicating they were deliberately extubated and then reintubated

Date of stopping/restarting intubation is shown by

*AIR.AccidentDate* (the date intubation stops) and *AIR.ReintuDate3 (t*he date it starts again)

Or

*AIR.DeliberateDate* (the date intubation stops) *and AIR.ReintuDate4* (the date it starts again)

Thus when the reintubation date is more than the stopping date +2, then the dates from stopdate+2 to reintubation date (but not including reintubation date) should be considered unintubated days. (Actually these are ‘not at risk days’, strictly speaking)

OR

SUPAIR will also contain information about episodes where intubation stops or starts

*SUPAIR.EventName*= Deliberate extubation *(*Database Value 1*)*

OR

*SUPAIR.EventName*= Accidental extubation (Database value 3)

Both show that there has been a stop in intubation, the date is given by *SUPAIR.SUPDate* for the corresponding row for that *SUPAIRPatientNo.*

Restarting of intubation is designated by *SUPAIR.EventName*= Reintubation (DB value 5)

So dates between *SUPAIR.SUPDate +2* ,corresponding to either *SUPAIR.EventName*= Deliberate extubation *(*Database Value 1*)* OR *SUPAIR.EventName*= Accidental extubation (Database value 3) and the *SUPAIR.SUPDate* corresponding to the next *SUPAIR.EventName*= Reintubation (DB value 5) will be considered NOT INTUBATED DAYS. (not including the end date and start date)

For tracheostomy the start date is

IF *ADM.NKQ* = tracheostomy, then this is latest of *ADM.Date1stIntu* OR *ENR.RandDate*

IF *ADM.NKQ* = mouth, then this is *AIR.TrachDate* if *AIR.IsTrach* = Yes

The day of tracheostomy is considered a trache day, not an ET day.

The stop date for trache will be the same as that given above for ET, with the same proviso that there may be breaks in it.

Thus we should have all the days from randomization to end of study labelled as either an ET day or a trache day or an unintubated day.

An intubated day is any day when the patient has either an ET day or a Trache day (or up to 2 days after these as long as this doesn't take them past the end of their time in ICU defined by the earlier of *FINISH.ICUDate* and *FINISH.CompleteDate)*

If we then label each day as either an antibiotic day or not, we can work out the proportion in each arm.

Antibiotic use per day is given in the ABTIME table. This contains only systematically administered antibiotics and antifungals that were prescribed from randomization to the end of the ICU portion of the study for the patient (end of ICU part of the study being actual discharge from ICU/death/withdrawal/90d follow up on ICU – see ICUendate below).

Antituberculous medicines have been excluded as have antiviral medications and topically administered antibiotics.

Here for each *ABTIME.PatientNo* there will be multiple episodes where antibiotics are started and stopped (the antibiotics designated by *ABTIME.MedName).*

To establish if an antibiotic is given on a given day, each antibiotic has a start and stop date.

Each antibiotic prescription is distinguished by *ABTIME.SeqNum* for each *ABTIME.PatientNo*

Of note. A day is defined as from 00:00 to 24:00, *ie* 24/2/19 00:00 is the same as 23/2/19 24:00.

Of note: the first day and the last day that antibiotics are given ‘count’ as antibiotic days.

Start date for each antibiotic *(ABTIME.MedName)*:

*ABTIME.FirstDate*

OR

If *ABTIME.OrFirst* = True then this indicates that the patient was on this at randomization (so *ABTIME.FirstDate* = *ENR.RandDate)*

End date for each antibiotic would use the earlier of the dates below:

*ABTIME.LastDate*

OR

If *ABTIME.OrLast* = True then this indicates that the patient was on this at discharge/death/withdrawal/90d follow up (so *ABTIME.LastDate* = ICUenddate*)*

ICUendate can be defined as follows:

Earliest of:

*ENR.RandDate +90* days (patient reached 90days follow up in ICU)

*FINISH.ICUDate* (patient was discharged from ICU or died there)

*FINISH.CompleteDate* (patient withdrew or was withdrawn)

So by comparing dates where antibiotics were given and dates where intubation was present the proportion of intubated days where antibiotics were administered can be calculated.

Where antibiotic dates were missing (start or end), these antibiotics have been excluded from the analysis. The number of antibiotic-patient episodes that were excluded will be listed as a footnote.

### ICU stay

This the length of time from *ENR.RandDate to* ICUendate

Patients that reached 90 days follow up in ICU are censored at that day.

Patients that are discharged to die or die in ICU are considered to have experienced the competing outcome ‘death’. Time of death is calculated by

*FINISH.ICUDate* IF *FINISH.OutCome =* Death (Database variable 1) or Home/transfer to Die (Database variable 2) AND {FINISH.HosDate = *FINISH.ICUDate* }

Note that the time of the competing risk is set at the time of discharge for those that are discharged to die because they are no longer at risk for the event of interest from that moment onwards. This time should not be interpreted as time of death.

### Ventilated Days

Patients will be considered as ventilated from the first point of ventilation until their last stopping of ventilation, breaks in ventilation will not be considered.

A complication could be if there are people who have ventilation stopped to allow them to go home to die. So if end of ventilation day = day of death or palliative discharge then we should consider this as a death for analysis.

Thus

If *FINISH.ICUDate = AIR.VentilationDate AND {FINISH.OutCome =* Death (Database variable 1) OR Home/transfer to Die (Database variable 2)} AND {FINISH.HosDate = *FINISH.ICUDate OR FINISH.HosSame =* True} then consider this as a death, rather than ventilation stop.

Then start is the **latest** of *ENR.RandDate* (Randomisation date)OR *AIR*.*StartDate* (Ventilation start date)

End of ventilation is the earliest of

1. 90 days after randomization.(*ENR.RandDate +90* days)
2. *FINISH.ICUDate* (Midday of Date of ICU discharge/death)
3. *FINISH.CompleteDate* (Midday of Date of withdrawl from the study.

Note that if *FINISH.IsCompleted* = 2 (“No, withdrew consent”) then we can only use the data if they permitted use of data up to this point, defined by *FINISH.IsAgree* = Yes. For other causes of *FINISH.IsCompleted* = No (values 3 or 4)we can use the data

1. *Stop ventilation time. This is the last (latest) time point at which ventilation is stopped and not followed by restarting ventilation.* Thus there may not be a stop ventilation time if either ventilation is never stopped or every time it is stopped it is restarted.

If there is no stop ventilation time then the end of ventilation is defined by *FINISH.ICUDate* or *FINISH.CompleteDate* if *FINISH.IsCompleted* =2 or 3 (withdrew) or 90 days after randomization

So stop ventilation time it is the latest of:

*AIR.VentilationDate* (date ventilation first stopped)

OR  *AIR.StopDate* (second time ventilation is stopped)

*OR*

*SUPAIR.SUPDate* for that patient (designated by *SUPAIR.PatientNo)* where *SUPAIR.EventName* = “*Ventilation stopped”* (Dbase value 2)

Which is not followed by one of the following

*AIR.ReintuDate5*

*Or*

*SUPAIR.SUPDate* for that patient (designated by *SUPAIR.PatientNo)* where *SUPAIR.EventName* = “*Ventilation restarted”* (Dbase value 4)

### Cost of ICU Stay

*FINISH. ICUCost + FINISH.EmergencyCost* gives the cost in Vietnamese Dong, this will be corrected to USD by dividing by 22, 660.83 (the exchange rate on 1/1/2018 as shown on <https://www.exchange-rates.org/Rate/USD/VND/1-1-2018> accessed 14/6/2019)

### Cost of ICU antibiotics

The cost of antibiotics prescribed during ICU stay for every participant are given in ABCOST. Each participant is denoted by their Patient Number (*ABCOST.PatientNo*) and each antibiotic by *ABCOST.SeqNum*

Thus for each participant the cost of ICU antibiotics is:

Sum of *ABCOST.cost* for every *ABCOST.SeqNum* for that *ABCOST.PatientNo*

This gives a cost in Vietnam Dong, converted to US dollars by dividing by 22, 660.83 (the exchange rate on 1/1/2018 as shown on <https://www.exchange-rates.org/Rate/USD/VND/1-1-2018> accessed 14/6/2019)

### Cost of hospital stay

*FINISH.InpatientCost*

This gives a cost in Vietnam Dong, converted to US dollars by dividing by 22, 660.83 (the exchange rate on 1/1/2018 as shown on <https://www.exchange-rates.org/Rate/USD/VND/1-1-2018> accessed 14/6/2019)

### ICU Mortality

This is the number of patients who died or were discharged palliatively during their first ICU stay/total randomised

Number who died during first ICU is the number of randomized participants in each arm for whom *FINISH.OutCome =* Death (Database variable 1) OR Home/transfer to Die (Database variable 2) AND {FINISH.HosDate = *FINISH.ICUDate*}

### Overall mortality; 28 and 90 Day mortality.

The best way to do this is to define the date of death (DOD) and date of censoring (by LTFU or withdrawal).

Defining Date of Death (DOD)

DOD may be the same as date of discharge (ICU or hospital) for those that die as inpatients in ICU or after discharge from ICU:

For these participants:

*FINISH.OutCome =* Death (Database variable 1)

The date of death is *FINISH.ICUDate if FINISH.ICUDate = FINISH.HosDate OR FINISH.HosSame =* True} (This is deaths in ICU)

OR

*FINISH.HosDate* if *FINISH.ICUDate* < *FINISH.HosDate*

A patient who dies at home may be found to have done so through the 28 day or 90 day follow up calls

For these

DOD = *FU.DeathDate28* if *FU.Status28* = Died

DOD *= FU_DAY90.DeathDate90* if *FU_DAY90.Status90* = Died

Then for 28 day mortality we count those where DOD - *ENR.RandDate* ≤ 28 days

For 90 day CFR we count those where DOD - *ENR.RandDate ≤ 90 days*

Censoring dates for patients who haven’t died:

For alive at 90 days, don't censor (this is the maximum follow up)

This group is defined by:

Maximum DAILY1n2.VSDate - *ENR.RandDate > 89 days* (still in ICU at day 90)

OR

*FINISH.ICUDate* - *ENR.RandDate > 89 days* (still in ICU at day 90)

OR

*FINISH.HosDate* - *ENR.RandDate > 89 days* (still in hospital at day 90)

OR

*FU_DAY90.IsSuccess90 =* Yes AND *FU_DAY90.Status90* = Alive

Of the remaining uncategorized patients, any patient who does not register as alive at 90 days (see above), or dead (note, this does not include palliate discharge) before 90 days has been lost to follow up or withdrew.

For these patients their censor date is

Date of withdrawal if *FINISH.IsCompleted* = 2 OR 3:

Date of withdrawal is indicated by *FINISH.CompleteDat*

Otherwise it is the latest of :

Date of 28 Day follow up call, if Alive at the time

Being alive at the call is identified by *FU.Status28* = Alive

Date of the call is identified by *FU.Date28*

OR

The last completed DAILY form

*Ie* the latest *DAILY1n2.VSDate* for that participant

OR

Date of hospital discharge - *FINISH.HosDate*

**Derivation of KM**

Start is *ENR.RandDate*

Death date is DOD

Censoring occurs as described above

Tetanus status is described by *ENR.IsTetanus*

**Table 3 Pre-specified subgroup analyses (for primary endpoint)**

|  | **Total** | **Intervention**  **N (%)** | **Control** | **Effect measure (95%CI)** | **p** |
| --- | --- | --- | --- | --- | --- |
| **Tetanus status** |  |  |  |  | ***** |
| **Tetanus** |  | **events/N (%)** | **events/N (%)** | **OR** |  |
| **Non-tetanus** |  | **events/N (%)** | **events/N (%)** | **OR** |  |
|  |  |  |  |  |  |
| **Prior intubation^a^** |  |  |  |  | ***** |
| **Intubation =<2 hours before randomisation** |  | **events/N (%)** | **events/N (%)** | **OR** |  |
| **Intubation >2 hours before randomisation** |  | **events/N (%)** | **events/N (%)** | **OR** |  |
|  |  |  |  |  |  |
| **Route of intubation^a^** |  |  |  |  | ***** |
| **ET Tube** |  | **Events/total follow-up days** | **Events/total follow-up days** | **Hazard ratio** |  |
| **Tracheostomy** |  | **Events/total follow-up days** | **Events/total follow-up days** | **Hazard ratio** |  |
|  |  |  |  |  |  |
| **Site^a^** |  |  |  |  | ***** |
| **NHTD** |  | **events/N (%)** | **events/N (%)** | **OR** |  |
| **HTD** |  | **events/N (%)** | **events/N (%)** | **OR** |  |
| **BVTV** |  | **events/N (%)** | **events/N (%)** | **OR** |  |

***Test for heterogeneity (i.e. the interaction term)**

**^a^ Adjusted for tetanus status**

Totals for ET Tube and Tracheostomy add up to more than sample size because some individuals received both types of intubation

## Derivation of Table 3

### Tetanus

*ENR.IsTetanus*

### Intubation <=2 hours before randomization

Time from intubation to randomization is given by:

*[ENR.RandDate + ENR. RandTime] - [ADM.Date1stIntu +ADM.Time1stIntu]*

Please note it is possible to be intubated after randomization (this counts as less than 2 hours before)

### ET Tube/Trache:

This will be analysed using a Cox regression analysis with endotracheal tube/tracheostomy tube as a time-dependent variable and adjusted for tetanus status as main effect.

**The date of primary endpoint is defined by:**

The first (earliest) occasion *(*dictated by the *HAIEVAL.HAIDate for each HIAEVAL.PatientNo)* when *(HAISUM.VAP*=True OR *HAISUM.VAT*=True)

Dates of ET/Tracheostomy have been defined on page 14

Site**:**

This analysis will be adjusted for tetanus as main effect

This is dictated by the first 3 characters of *ENR.PatientNo*:

020 = NHTD

003= HTD

103= BVTV

**Table 4. Safety Analysis**

|  | **Intervention**  **N=** | **Control**  **N=** | **effect measure**  **(95% CI)** | **p** |
| --- | --- | --- | --- | --- |
| **Total Days on ECMO** |  |  |  |  |
| **At least one day of ECMO** |  |  | **OR** |  |
| **Total days with haemodynamic support** |  |  |  |  |
| **At least one day with haemodynamic support** |  |  | **OR** |  |
| **Total days with renal failure/renal support** |  |  |  |  |
| **At least one day with renal failure/renal support** |  |  | **OR** |  |
| **Total days with DIC** |  |  |  |  |
| **At least one day with DIC** |  |  | **OR** |  |
| **Total days with transfusion** |  |  |  |  |
| **At least one day with transfusion** |  |  | **OR** |  |
| **Total days with potential tracheal complication** |  |  |  |  |
| **At least one day with potential tracheal complication** |  |  | **OR** |  |
| **Total Grade 3/4 AEs** |  |  |  |  |
| **at least one Grade 3/4 AE** |  |  | **OR** |  |
| **Trachea related complications at 28 days** |  |  |  |  |
| **Trachea related complications at 90 days** |  |  |  |  |

The “Total” variables are summarized as mean, median and 1^st^/3^rd^ quartile. The binary variables are summarized as number with event (percentage).

## Derivation of Table 4

The frequency of adverse events will be summarized (in terms of both the total number of days with events and the number of patients with at least one day with an event). The proportion of patients with at least one adverse event (overall and for each specific event separately) will be summarized and compared between the two treatment groups; we use logistic regression for the confidence intervals. P-values are based on the chi-square test for independence in case the expected number of events in each cell is at least 1, and Fisher’s exact test otherwise.

**Total Days on ECMO (mean) and At least one day of ECMO**

*DAILY1n2.ECMO*

**Total days with haemodynamic support and At least one day with haemodynamic support**

*DAILY1n2.Haemodynamic*

**Total days with renal failure/renal support and At least one day with renal failure/renal support**

*DAILY1n2.Renal*

**Total days with DIC and At least one day with DIC**

*DAILY1n2.DIC*

**Total days with transfusion and At least one transfusion (this will be done for all patients and according to tetanus status. Results stratified by tetanus will be presented in text, not in a table)**

*DAILY1n2.Transfusion*

**Total days with potential tracheal complication and At least one day with potential tracheal complication**

*DAILY1n2.Tracheal*

### Total Grade 3/4 AEs

This is the sum of all event-days where any of these were present, if a patient has multiple events on the same day, each event will count (eg. Patient with ECMO, Haemodynamic and renal on the same day registers 3 event-days for that day)

*DAILY1n2.ECMO*

*DAILY1n2.Haemodynamic*

*DAILY1n2.Renal*

*DAILY1n2.DIC*

*DAILY1n2.Transfusion*

*DAILY1n2.Tracheal*

*DAILY1n2.Grade*

### Number with at least one Grade 3/4 AE

Patient with an occurrence of any one of the below will be summed for each arm and compared

*DAILY1n2.ECMO*

*DAILY1n2.Haemodynamic*

*DAILY1n2.Renal*

*DAILY1n2.DIC*

*DAILY1n2.Transfusion*

*DAILY1n2.Tracheal*

*DAILY1n2.Grade*

**Trachea related complications at 28 days in survivors not lost to follow up**

If any of the below are present the patient is considered to have a tracheal related complication at 28 days:

*FU.IsStridor6*

*FU.IsBleeding6*

*FU.IsTracheo6*

*FU.IsOther6*

**Trachea related complications at 90 days in survivors not lost to follow up**

If any of the below are present the patient is considered to have a tracheal related complication at 90 days:

*FU_DAY90.IsStridor12*

*FU_DAY90.IsBleeding12*

*FU_DAY90.IsTracheo12*

*FU_DAY90.IsOther12*

**Supplementary material: analysis of blood transfusion**

During the DSMB meetings it became clear that there was a significant difference between the two arms in terms of the proportion of patients that were requiring transfusion of blood products. The DSMB felt that there was no evidence that this related to the intervention. In order to explore this further we additionally compare the following data from the Transfusion form between arms both in total and according to tetanus status:

Total receiving any blood products

Designated by *TRANSF.PaRecBlood* (Yes/No)

Total receiving RBC;

Designated by *TRANSF.RBC* (Yes/No)

Total receiving Platelets;

Designated by *TRANSF.Platelet* (Yes/No)

Total receiving cryoprecipitate

Designated by *TRANSF.CryoPre* (Yes/No)

Total receiving plasma

Designated by *TRANSF.Plasma* (Yes/No)

Total Receiving any one of Platelets/cryoprecipitate/plasma

Designated by any of

*TRANSF.Platelet* =yes OR *TRANSF.CryoPre* = yes *TRANSF.Plasma* = yes

All these differences are reported as odds ratios and differences are tested for using likelihood ratio tests.

Total volume of RBC transfused

Designated by

(*TRANSF.RBCNB1* * *TRANSF.RBCRS1) +* (*TRANSF.RBCNB2* * *TRANSF.RBCRS2) +* (*TRANSF.RBCNB3* * *TRANSF.RBCRS3)* in mL

In the analysis of total volume, we exclude the individuals that didn’t receive any RBC (TRANSF.RBC=No). We use the Box-Cox procedure with intervention arm and tetanus status as covariables to find a suitable transformation. If reasonable, we use the identity or log transformation. After the transformation, arms are compared using linear regression. As effect measure we report the difference in expected value on the transformed scale (and report the transformation we used). Since this may be hard to interpret, we also plot the distribution of the variable by intervention arm and tetanus status via histograms. We add to the histograms the mean value with 95% confidence interval for each combination of intervention arm and tetanus status.

**Table 5 Analysis of Blood Transfusion (supplementary table)**

|  | **Intervention**  **N=** | **Control**  **N=** | **Effect measure (95% CI)** | **p-value** |
| --- | --- | --- | --- | --- |
| **Total receiving any blood products** |  |  |  |  |
| **Total receiving RBC** |  |  |  |  |
| **Total receiving platelets** |  |  |  |  |
| **Total receiving cryoprecipitates** |  |  |  |  |
| **Total receiving plasma** |  |  |  |  |
| **Total receiving any one of platelets, cryo, plasma** |  |  |  |  |
| **Total volume of RBC** |  |  |  |  |
| **Patients with tetanus** |  |  |  |  |
| **Number receiving any blood products** |  |  |  |  |
| **Number receiving RBC** |  |  |  |  |
| **Number receiving platelets** |  |  |  |  |
| **Number receiving cryoprecipitates** |  |  |  |  |
| **Number receiving plasma** |  |  |  |  |
| **Number receiving any one of platelets, cryo, plasma** |  |  |  |  |
| **Volume of RBC** |  |  |  |  |
| **Patients without tetanus** |  |  |  |  |
| **Number receiving any blood products** |  |  |  |  |
| **Number receiving RBC** |  |  |  |  |
| **Number receiving platelets** |  |  |  |  |
| **Number receiving cryoprecipitates** |  |  |  |  |
| **Number receiving plasma** |  |  |  |  |
| **Number receiving any one of platelets, cryo, plasma** |  |  |  |  |
| **Volume of RBC** |  |  |  |  |
