# Supplementary material for: Effectiveness of Continuous Endotracheal Cuff Pressure Control for the Prevention of Ventilator-Associated Respiratory Infections: An Open-Label Randomized, Controlled Trial
Source: Clin Infect Dis. 2021 Aug 22;74(10):1795–803. doi: 10.1093/cid/ciab724 (PMC9155610; doi:10.1093/cid/ciab724)
Supplement: ciab724_suppl_Supplementary_Tables_and_Figure [file ciab724_suppl_supplementary_tables_and_figure.docx]

**Supplementary Figure 1 showing the maintenance of targeted cuff pressure among the study participants.**

Cuff pressure was recorded 8 hourly in all patients. Plot shows estimated probability of cuff pressure falling within target range.


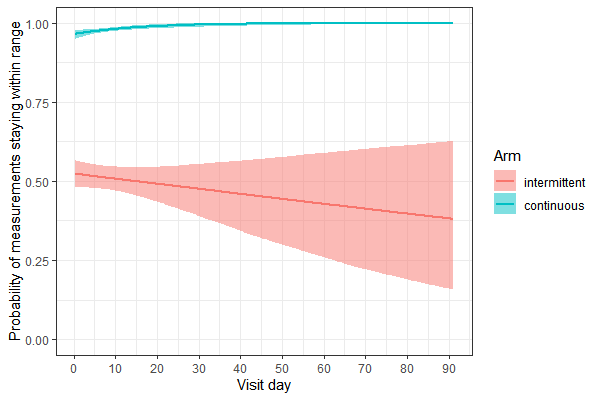


**Supplementary Table 1. The frequency of extubation and reintubation events**

| Characteristic | Intermittent cuff pressure control (N =186) | Intermittent cuff pressure control (N = 222) |
| --- | --- | --- |
| Accidental extubation | 19/186 (10.2%) | 30/222 (13.5%) |
| Deliberate self-extubation | 129/186 (69.4%) | 137/222 (61.7%) |
| Reintubation | 38/186 (20.4%) | 55/222 (24.8%) |

**Supplementary Table 2. The frequency of microorganisms isolated in patients with microbiologically-confirmed VAP**

| Pathogens | All aetiology (N=144) | Intermittent cuff pressure control (N=67) | Continuous cuff pressure control (N = 77) |
| --- | --- | --- | --- |
| *Pseudomonas aeruginosa* | 40 (27.8%) | 17 (25.4%) | 23 (29.9%) |
| *Acinetobacter baumannii* | 31 (21.5%) | 16 (23.9%) | 15 (19.5%) |
| *Klebsiella pneumoniae* | 29 (20.1%) | 16 (23.9%) | 13 (16.9%) |
| *Staphylococcus aureus* | 13 (9.0%) | 6 (9.0%) | 7 (9.1%) |
| *Haemophilus influenzae* | 7 (4.9%) | 2 (3.0%) | 5 (6.5%) |
| *Streptococcus pneumoniae* | 6 (4.2%) | 2 (3.0%) | 4 (5.2%) |
| *Streptococcus mitis* | 3 (2.1%) | 1 (1.5%) | 2 (2.6%) |
| *Stenotrophomonas maltophilia* | 3 (2.1%) | 1 (1.5%) | 2 (2.6%) |
| *Acinetobacter nosocomialis* | 2 (1.4%) | 1 (1.5%) | 1 (1.3%) |
| *Escherichia coli* | 2 (1.4%) | 2 (3.0%) | 0 (0.0%) |
| *Candida albicans* | 2 (1.4%) | (0.0%) | 2 (2.6%) |
| *Haemophilus parainfluenzae* | 2 (1.4%) | 1 (1.5%) | 1 (1.3%) |
| *Corynebacterium striatum* | 1 (0.7%) | 1 (1.5%) | (0.0%) |
| *Aspergillus fumigatus* | 1 (0.7%) | (0.0%) | 1 (1.3%) |
| *Elizabethkingia miricola* | 1 (0.7%) | (0.0%) | 1 (1.3%) |
| *Moraxella catarrhalis* | 1 (0.7%) | 1 (1.5%) | (0.0%) |

**Supplementary Table 3. The frequency of microorganisms isolated in patients with** microbiologically-confrimed VAT

| Pathogens | All aetiology (N=68) | Intermittent cuff pressure control (N=30) | Continuous cuff pressure control (N = 38) |
| --- | --- | --- | --- |
| *Pseudomonas aeruginosa* | 17 (25.0%) | 8 (26.7%) | 9 (23.7%) |
| *Acinetobacter baumannii* | 16 (23.5%) | 6 (20.0%) | 10 (26.3%) |
| *Staphylococcus aureus* | 11 (16.2%) | 4 (13.3%) | 7 (18.4%) |
| *Klebsiella pneumoniae* | 5 (7.4%) | 1 (3.3%) | 4 (10.5%) |
| *Serratia marcescens* | 3 (4.4%) | 2 (6.7%) | 1 (2.6%) |
| *Stenotrophomonas maltophilia* | 3 (4.4%) | 1 (3.3%) | 2 (5.3%) |
| *Candida tropicalis* | 1 (1.5%) | (0.0%) | 1 (2.6%) |
| *Corynebacterium striatum* | 1 (1.5%) | 1 (3.3%) | (0.0%) |
| *Escherichia coli* | 1 (1.5%) | 0 (0.0%) | 1 (2.6%) |
| *Streptococcus oralis* | 1 (1.5%) | 1 (3.3%) | (0.0%) |
| *Citrobacter koseri* | 1 (1.5%) | (0.0%) | 1 (2.6%) |
| *Burkholderia cepacia* | 1 (1.5%) | 1 (3.3%) | (0.0%) |
| *Haemophilus influenzae* | 1 (1.5%) | 1 (3.3%) | (0.0%) |
| *Elizabethkingia meningoseptica* | 1 (1.5%) | (0.0%) | 1 (2.6%) |
| *Pasteurella multocida* | 1 (1.5%) | 1 (3.3%) | (0.0%) |
| *Streptococcus mitis* | 1 (1.5%) | 1 (3.3%) | (0.0%) |
| *Haemophilus haemolyticus* | 1 (1.5%) | 1 (3.3%) | (0.0%) |
| *Enterobacter cloacae* | 1 (1.5%) | 1 (3.3%) | (0.0%) |
| *Streptococcus anginosus* | 1 (1.5%) | (0.0%) | 1 (2.6%) |
